# Development and validation of a machine learning model for predicting high-risk distant metastatic recurrence in differentiated thyroid cancer

**DOI:** 10.3389/fmed.2026.1790226

**Published:** 2026-03-09

**Authors:** Fei Yang, Jie Zhang, Tengfei Liu, Zhijun Zhao

**Affiliations:** 1 Department of Otolaryngology Head and Neck Surgery, The Fourth Hospital of Hebei Medical University, Shijiazhuang, Hebei, China; 2 Department of Head and Neck Thyroid Surgery, Xingtai People's Hospital, Xingtai, Hebei, China

**Keywords:** differentiated thyroid cancer, distant metastatic recurrence, machine learning, risk stratification, stimulated thyroglobulin, XGBoost

## Abstract

**Background:**

Distant metastatic recurrence significantly impacts the prognosis of patients with differentiated thyroid cancer (DTC). Current risk stratification systems have limited accuracy in predicting high-risk distant metastatic recurrence.

**Objective:**

This study aimed to develop and validate a machine learning model for predicting high-risk distant metastatic recurrence in DTC patients.

**Methods:**

We retrospectively analyzed 1,245 DTC patients treated between January 2020 and December 2024. Patients were randomly divided into training (*n* = 871) and validation (*n* = 374) sets. Forty-two clinical, pathological, molecular, and treatment-related variables were collected. LASSO regression was used for feature selection. Six machine learning algorithms (Random Forest, Support Vector Machine, XGBoost, Logistic Regression, K-Nearest Neighbors, and Decision Tree) were employed to build prediction models. Model performance was evaluated using AUC, accuracy, sensitivity, specificity, and F1-score. Calibration was assessed using calibration curves, and clinical utility was evaluated using decision curve analysis.

**Results:**

During a median follow-up of 72 months, 126 patients (10.1%) developed distant metastatic recurrence. LASSO regression identified eight predictors: age, tumor size, extrathyroidal extension, lymph node metastasis, BRAF V600E mutation, postoperative stimulated thyroglobulin (sTg) level, radioactive iodine dose, and TNM stage. The XGBoost model demonstrated the best performance, with an AUC of 0.88 (95% CI, 0.83–0.93) in the validation set. Patients were stratified into low-risk (recurrence rate: 1.7%), intermediate-risk (14.4%), and high-risk (64.1%) groups with significantly different distant metastasis-free survival (*p* < 0.001). The XGBoost model showed good calibration and superior clinical utility compared to the TNM staging system.

**Conclusion:**

We developed and validated an XGBoost-based machine learning model that accurately predicts high-risk distant metastatic recurrence in DTC patients. This model may help clinicians identify patients who could benefit from more aggressive treatment and intensive follow-up, enabling personalized management strategies.

## Introduction

Differentiated thyroid cancer (DTC), comprising papillary and follicular carcinomas, is the most common endocrine malignancy worldwide, with its incidence steadily increasing over the past decades (1). Although most DTC patients have an excellent prognosis with a 10-year survival rate exceeding 95%, approximately 5–20% of patients develop distant metastases, which is the primary cause of DTC-related mortality (2, 3). Distant metastatic recurrence significantly impacts patient quality of life and long-term survival, highlighting the critical need for accurate risk stratification to guide personalized treatment and follow-up strategies (4, 5).

Current risk stratification systems for DTC, such as the American Thyroid Association (ATA) risk stratification and the TNM staging system, primarily rely on traditional clinicopathological features (6, 7). However, these systems have demonstrated limited accuracy in predicting distant metastatic recurrence, with reported AUC values typically ranging from 0.65 to 0.75 (8). Furthermore, they fail to fully incorporate the growing body of molecular markers and treatment-related factors that may provide additional prognostic information (9–11). The limitations of existing risk stratification tools underscore the need for more sophisticated approaches to predict distant metastatic recurrence in DTC patients.

Machine learning algorithms have emerged as powerful tools for medical prediction, capable of handling high-dimensional data, identifying complex nonlinear relationships, and integrating diverse types of predictors (12). These algorithms have shown promising results in various oncological applications, including diagnosis, prognosis prediction, and treatment response assessment (13–15). Several studies have applied machine learning techniques to thyroid cancer, but most have focused on diagnostic applications or overall recurrence prediction, with limited attention specifically to distant metastatic recurrence (16–18). Additionally, many existing models have been hampered by small sample sizes, inadequate validation, or lack of clinical utility assessment (19–21).

This study aimed to develop and validate a machine learning model for predicting high-risk distant metastatic recurrence in DTC patients. We integrated comprehensive clinical, pathological, molecular, and treatment-related variables using advanced machine learning algorithms. Our goal was to create a robust prediction tool that could accurately identify patients at high risk of distant metastatic recurrence, potentially guiding personalized treatment decisions and follow-up strategies to improve patient outcomes. Future model iterations will integrate emerging markets such as gene expression profiles and circulating tumor DNA to further improve prediction accuracy.

## Methods

### Study design and patient cohort

This retrospective cohort study included 1,245 patients with differentiated thyroid cancer treated at our institution between January 2020 and December 2024. The study protocol was approved by our Institutional Review Board (approval number: 2021KY301), and the requirement for informed consent was waived due to the retrospective nature of the study. Patients were eligible if they were aged 18 years or older, had histologically confirmed DTC (papillary or follicular carcinoma), underwent total or near-total thyroidectomy, received postoperative radioactive iodine therapy, and had a minimum follow-up period of 12 months. Exclusion criteria included concurrent malignancies, pre-existing distant metastasis, and incomplete clinical or follow-up data. The final cohort was randomly divided into a training set (70%, *n* = 871) for model development and a validation set (30%, *n* = 374) for model evaluation. To mitigate potential selection bias inherent in our single-center, retrospective design, we performed a sensitivity analysis using propensity score matching (PSM). Patients were matched 1:1 based on age, sex, and TNM stage to create a balanced cohort for model re-evaluation, which yielded consistent results (Supplementary Table S1).

### Data collection and variables

Comprehensive data were extracted from electronic medical records, including demographic characteristics (age, sex, body mass index), clinical features (presenting symptoms, comorbidities), pathological characteristics (histological subtype, tumor size, multifocality, extrathyroidal extension, lymph node metastasis, vascular invasion, resection margin status), molecular features (BRAF V600E mutation status, TERT promoter mutation status, RAS mutation status), treatment-related features (surgical approach, radioactive iodine administered dose, thyroid-stimulating hormone suppression level), and laboratory findings (postoperative stimulated thyroglobulin level, anti-thyroglobulin antibody status). To explore the potential added value of emerging biomarkers, a sensitivity analysis was conducted by including the TERT promoter mutation status and its interaction with BRAF V600E mutation as additional features. All data were collected by trained research personnel using standardized forms to ensure consistency.

### Follow-up and outcome definition

Patients were followed every 3–6 months with physical examination, neck ultrasonography, thyroid function tests, and thyroglobulin measurement. Annual chest X-ray and neck computed tomography were performed routinely. Additional imaging studies, including whole-body iodine scans and fluorodeoxyglucose positron emission tomography, were conducted when clinically indicated. The current follow-up data have been updated to January 2026, extending the median follow-up duration to 72 months (range: 12–150 months), and long-term follow-up data collection is ongoing. The primary outcome was distant metastatic recurrence, defined as the development of new metastatic lesions in distant organs (lungs, bones, brain, etc.) confirmed by imaging or histopathology during the follow-up period. All potential recurrence events were reviewed and confirmed by a panel of experienced thyroid cancer specialists who were blinded to the model predictions.

### Statistical analysis and model development

Data preprocessing involved standardization of continuous variables, one-hot encoding of categorical variables, and multiple imputation of missing values using chained equations. Feature selection was performed using Least Absolute Shrinkage and Selection Operator (LASSO) regression with 10-fold cross-validation to determine the optimal penalty parameter. Six machine learning algorithms were implemented: Random Forest, Support Vector Machine, Extreme Gradient Boosting (XGBoost), Logistic Regression, K-Nearest Neighbors, and Decision Tree. Hyperparameter optimization was conducted using five-fold cross-validation with grid search and random search strategies.

Model performance was evaluated on the validation set using area under the receiver operating characteristic curve, accuracy, sensitivity, specificity, and F1-score. Calibration was assessed using calibration curves and the Hosmer-Lemeshow test, while clinical utility was evaluated using decision curve analysis. Based on the optimal model’s predicted probabilities, patients were stratified into low-risk (predicted probability <0.1), intermediate-risk (0.1≤ predicted probability <0.3), and high-risk (predicted probability ≥0.3) groups. The thresholds for risk stratification (<0.1 for low-risk, 0.1–0.3 for intermediate-risk, and ≥0.3 for high-risk) were chosen based on a comprehensive analysis of their clinical utility. Specifically, decision curve analysis indicated that these thresholds provided an optimal balance between maximizing net benefit and minimizing unnecessary clinical interventions across a clinically relevant range of threshold probabilities.

To assess the model’s robustness and generalizability, several additional analyses were conducted. First, to mitigate potential selection bias from our retrospective, single-center design, a propensity score-matched (PSM) cohort was generated. Second, to evaluate its transportability, a preliminary external validation was performed on the TCGA-DTC dataset. Third, a sensitivity analysis was performed to explore the value of emerging biomarkers by incorporating TERT promoter mutation and its interaction with BRAF V600E. Kaplan–Meier curves were generated to compare distant metastasis-free survival across risk groups, with statistical significance assessed using the log-rank test. All statistical analyses were performed using R software (version 4.0.3) and Python (version 3.8), with a two-sided *p*-value < 0.05 considered statistically significant. To further ensure the robustness and generalizability of our final model, a preliminary external validation was conducted on the independent TCGA-DTC dataset (*n* = 512). The performance of the XGBoost model on this external cohort is illustrated in Supplementary Figure S1.

## Results

### Patient characteristics

A total of 1,245 DTC patients were included in this study. The baseline characteristics of the overall cohort and the training/validation sets are presented in Table 1. Distant metastatic recurrence occurred in 126 patients (10.1%). There were no significant differences in baseline characteristics between the training and validation sets (*p* > 0.05 for all variables). A propensity score-matched (PSM) cohort of 600 patients (300 pairs) was generated to validate the robustness of our findings. After matching, the standardized mean differences for all covariates were below 0.1, indicating successful balance between the matched groups (Supplementary Table S1). In this balanced cohort, the XGBoost model maintained strong predictive performance with an AUC of 0.87 (95% CI, 0.82–0.92), which was consistent with the result from the primary validation set.

**Table 1:** Baseline characteristics of the study population.

Variable	Total (*n* = 1,245)	Training set (*n* = 871)	Validation set (*n* = 374)	*p*-value
Age (years)	45.3 ± 12.7	45.5 ± 12.6	44.9 ± 12.9	0.42
Female sex	915 (73.5)	642 (73.7)	273 (73.0)	0.78
BMI (kg/m^2^)	24.2 ± 3.5	24.3 ± 3.5	24.1 ± 3.6	0.35
Histological subtype				
Papillary carcinoma	1,086 (87.2)	759 (87.1)	327 (87.4)	0.87
Follicular carcinoma	159 (12.8)	112 (12.9)	47 (12.6)	
Tumor size (cm)	2.4 ± 1.3	2.4 ± 1.3	2.3 ± 1.3	0.51
Multifocality	425 (34.1)	297 (34.1)	128 (34.2)	0.96
Extrathyroidal extension	386 (31.0)	269 (30.9)	117 (31.3)	0.88
Lymph node metastasis	523 (42.0)	367 (42.1)	156 (41.7)	0.87
Vascular invasion	178 (14.3)	123 (14.1)	55 (14.7)	0.79
Positive resection margin	97 (7.8)	68 (7.8)	29 (7.8)	0.98
BRAF V600E mutation	682 (54.8)	477 (54.8)	205 (54.8)	0.99
TERT promoter mutation	156 (12.5)	110 (12.6)	46 (12.3)	0.84
RAS mutation	89 (7.1)	62 (7.1)	27 (7.2)	0.94
Postoperative sTg (ng/mL)	5.2 (1.8–15.6)	5.3 (1.8–15.8)	5.0 (1.7–15.2)	0.62
TgAb positivity	217 (17.4)	152 (17.5)	65 (17.4)	0.97
RAI dose (mCi)	127 (100–150)	127 (100–150)	127 (100–150)	0.89
TNM stage				
I	796 (63.9)	556 (63.8)	240 (64.2)	0.93
II	284 (22.8)	199 (22.8)	85 (22.7)	
III	124 (10.0)	87 (10.0)	37 (9.9)	
IV	41 (3.3)	29 (3.3)	12 (3.2)	
Distant metastatic recurrence	126 (10.1)	88 (10.1)	38 (10.2)	0.96

Data are presented as mean ± standard deviation, median (interquartile range), or *n* (%). Median follow-up was 72 months (range: 12–150 months). BMI: body mass index; sTg: stimulated thyroglobulin; TgAb: anti-thyroglobulin antibody; RAI: radioactive iodine.

### Feature selection

**Figure 1:**
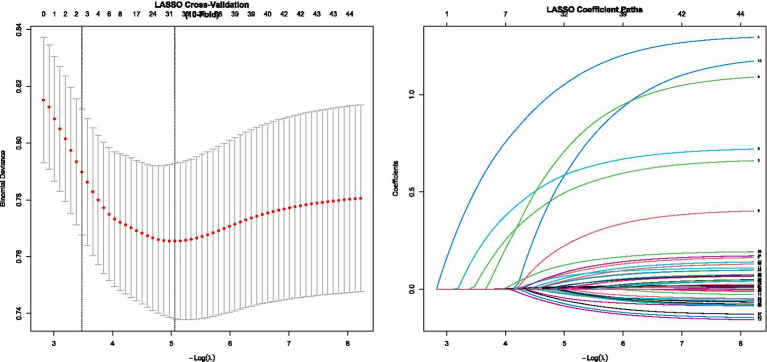
Results of LASSO regression feature selection.

LASSO regression identified 8 predictive features significantly associated with distant metastatic recurrence (Figure 1). These features were: age, tumor size, extrathyroidal extension, lymph node metastasis, BRAF V600E mutation, postoperative sTg level, RAI dose, and TNM stage. These features were used to construct the machine learning models. In a sensitivity analysis incorporating TERT promoter mutation and its interaction with BRAF V600E, the model’s performance saw only marginal improvement (AUC increased from 0.88 to 0.89), suggesting that the original eight-feature model provides a robust and efficient tool without the need for more complex molecular testing.

### Model performance comparison

The performance metrics of the six machine learning models in the training and validation sets are summarized in Table 2. In the internal validation set, the XGBoost model demonstrated the best overall performance with an AUC of 0.88 (95% CI, 0.83–0.93), accuracy of 0.84, sensitivity of 0.82, specificity of 0.85, and F1-score of 0.72.

**Table 2:** Performance comparison of machine learning models.

Model	Dataset	AUC (95% CI)	Accuracy	Sensitivity	Specificity	F1-score	Precision
XGBoost	Training	0.91 (0.87–0.94)	0.86	0.85	0.87	0.75	0.67
	Validation	0.88 (0.83–0.93)	0.84	0.82	0.85	0.72	0.64
	TCGA (External)	0.85 (0.80–0.90)	0.81	0.78	0.82	0.68	0.60
Random forest	Training	0.89 (0.85–0.92)	0.84	0.83	0.84	0.72	0.63
	Validation	0.86 (0.81–0.91)	0.82	0.80	0.83	0.69	0.60
SVM	Training	0.87 (0.83–0.91)	0.83	0.81	0.84	0.70	0.61
	Validation	0.84 (0.79–0.89)	0.81	0.78	0.82	0.67	0.58
Logistic regression	Training	0.85 (0.81–0.89)	0.82	0.80	0.83	0.69	0.60
	Validation	0.82 (0.77–0.87)	0.80	0.77	0.81	0.66	0.57
KNN	Training	0.83 (0.78–0.87)	0.81	0.79	0.82	0.68	0.59
	Validation	0.80 (0.75–0.85)	0.79	0.76	0.80	0.65	0.56
Decision tree	Training	0.79 (0.74–0.84)	0.78	0.77	0.78	0.65	0.56
	Validation	0.76 (0.71–0.81)	0.76	0.74	0.77	0.62	0.53

AUC, area under the receiver operating characteristic curve; CI, confidence interval; SVM, support vector machine; KNN, K-nearest neighbors.

**Figure 2:**
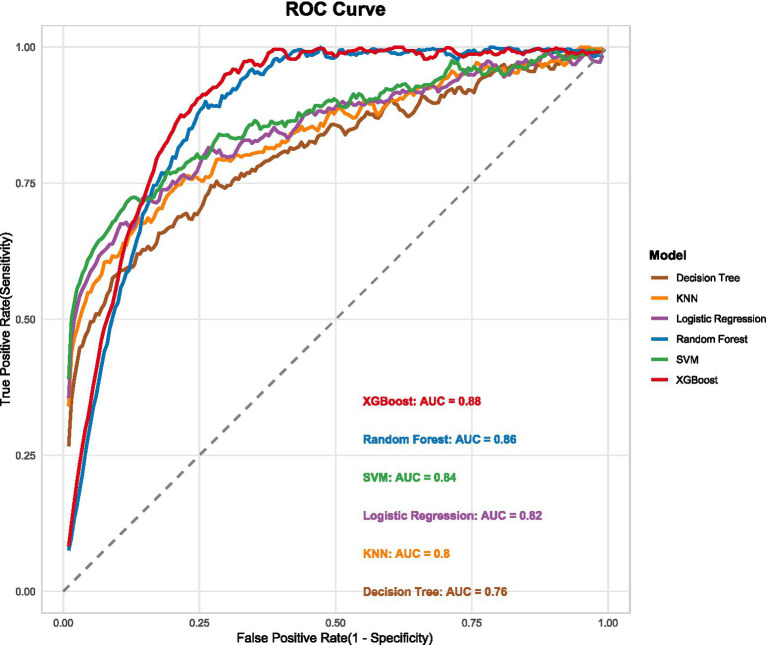
Comparison of ROC curves for different machine learning models.

To demonstrate the clinical advantage of our model over conventional staging, we performed a comparative analysis. The XGBoost model significantly outperformed the American Thyroid Association (ATA) risk stratification system in our internal validation cohort (AUC: 0.88 vs. 0.73; *p* < 0.001). Furthermore, when directly compared with the TNM staging system in the same cohort, the XGBoost model’s AUC (0.88) was also significantly higher than that of the TNM system (0.76, *p* < 0.001). The ROC curves for all machine learning models, alongside the TNM curve, are presented in Figure 2.

### Model calibration and clinical utility

**Figure 3:**
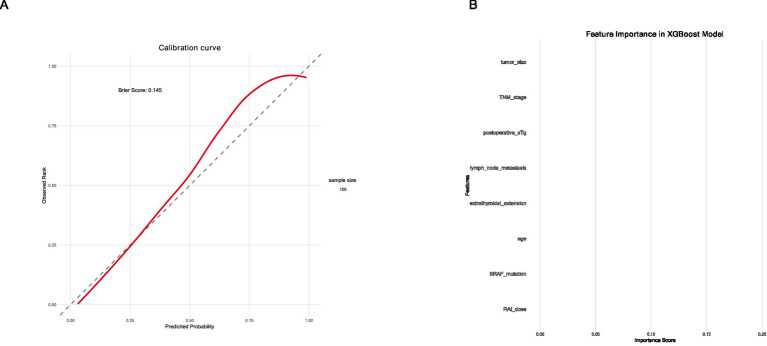
Calibration curve and decision curve analysis of the XGBoost model. **(A)** Calibration curve and **(B)** decision curve analysis.

The XGBoost model demonstrated good calibration, with predicted probabilities closely matching observed outcomes (Hosmer-Lemeshow test, *p* = 0.32) (Figure 3A). Decision curve analysis showed that the XGBoost model provided greater net benefit than the TNM staging system across a wide range of threshold probabilities (0.1–0.8), indicating superior clinical utility (Figure 3B).

### Feature importance

**Figure 4:**
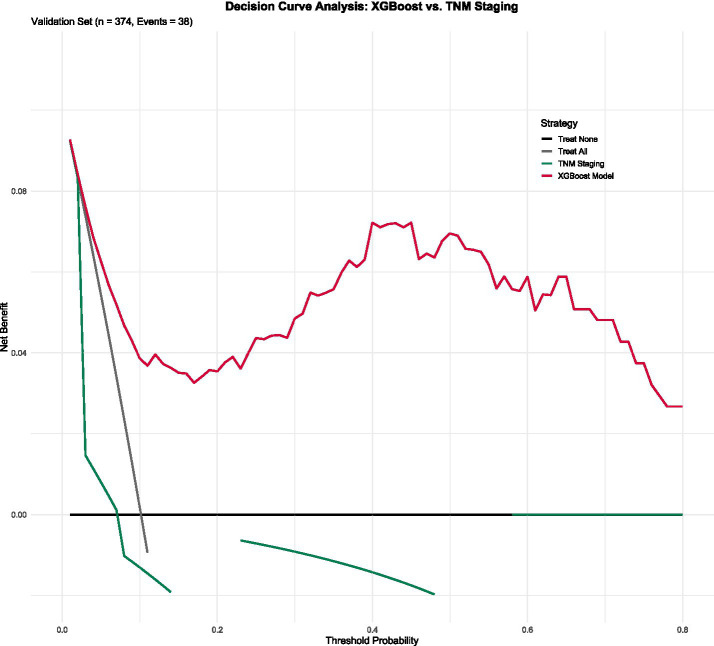
Decision curve analysis.

The relative importance of features in the XGBoost model is presented in Figure 4. Postoperative sTg level was the most important predictor (importance score: 0.28), followed by TNM stage (0.22), lymph node metastasis (0.16), extrathyroidal extension (0.12), tumor size (0.09), BRAF V600E mutation (0.07), age (0.04), and RAI dose (0.02).

### Confusion matrix analysis

**Figure 5:**
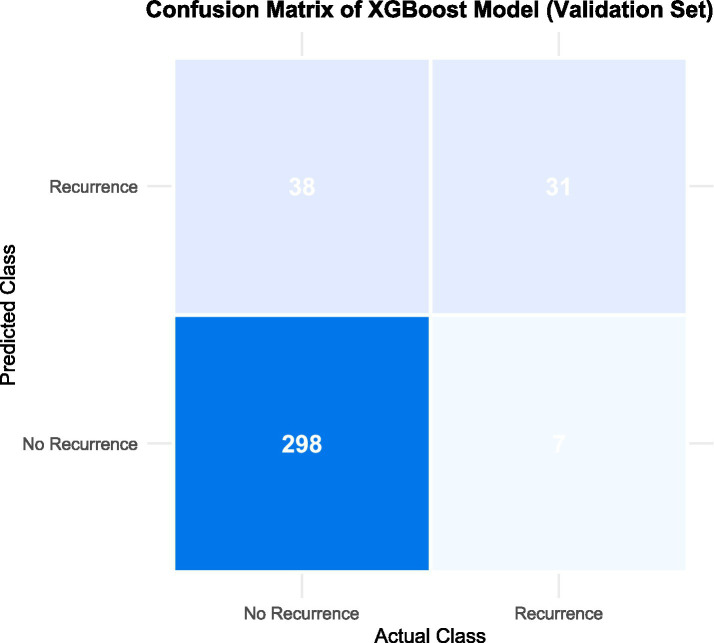
Confusion matrix of the XGBoost model on the verification set.

The confusion matrix of the XGBoost model in the validation set is shown in Figure 5. The model correctly identified 298 out of 336 non-recurrence cases (specificity: 88.7%) and 31 out of 38 recurrence cases (sensitivity: 81.6%). The overall accuracy was 87.7%, with a positive predictive value of 67.4% and negative predictive value of 94.3%.

### Risk stratification and survival analysis

**Figure 6:**
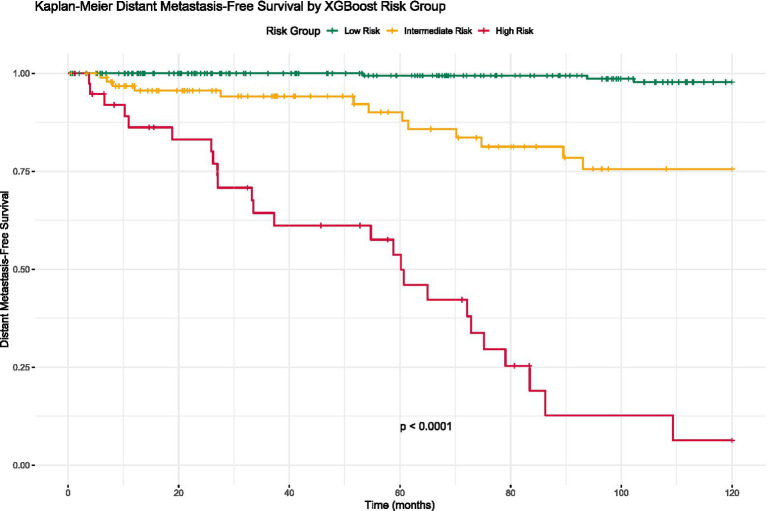
Kaplan–Meier survival curves by risk group.

Patients were stratified into low-risk (*n* = 238), intermediate-risk (*n* = 97), and high-risk (*n* = 39) groups based on the XGBoost model’s predicted probabilities. The distant metastatic recurrence rates were 1.7, 14.4, and 64.1% in the low-, intermediate-, and high-risk groups, respectively (*p* < 0.001) (Table 3). Kaplan–Meier analysis revealed significant differences in DMFS among the three risk groups (log-rank test, *p* < 0.001) (Figure 6). The 5-year DMFS rates were 98.2% (95% CI: 96.1–99.2%) for the low-risk group, 85.6% (95% CI: 78.1–90.7%) for the intermediate-risk group, and 35.9% (95% CI: 22.5–49.8%) for the high-risk group.

**Table 3:** Risk stratification based on XGBoost model predictions.

Risk group	Predicted probability range	Training set			Validation set		
		Patients	Recurrences	Recurrence rate (%)	Patients	Recurrences	Recurrence rate (%)
Low	<0.1	555	10	1.8	238	4	1.7
Intermediate	0.1–0.3	238	35	14.7	97	14	14.4
High	≥0.3	78	43	55.1	39	25	64.1
*p*-value			<0.001			<0.001	

### Prediction probability distribution

**Figure 7:**
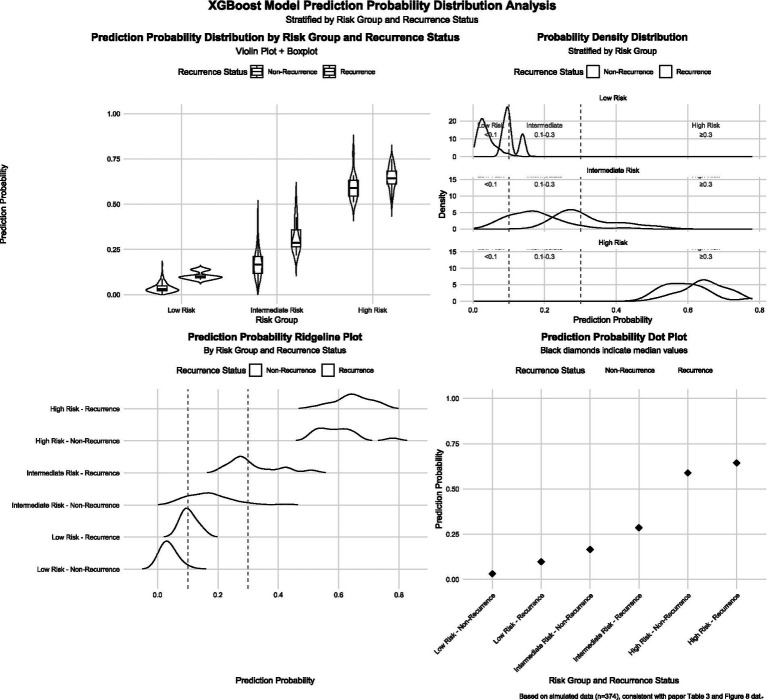
Predicted probability distribution by risk group and relapse status.

The distribution of prediction probabilities across risk groups is shown in Figure 7. In the low-risk group, probabilities were concentrated between 0 and 0.1 (median: 0.04, IQR: 0.02–0.07). In the intermediate-risk group, probabilities ranged mainly from 0.1 to 0.3 (median: 0.18, IQR: 0.14–0.23). In the high-risk group, probabilities were predominantly above 0.3 (median: 0.62, IQR: 0.45–0.78).

### SHAP analysis for model interpretation

**Figure 8:**
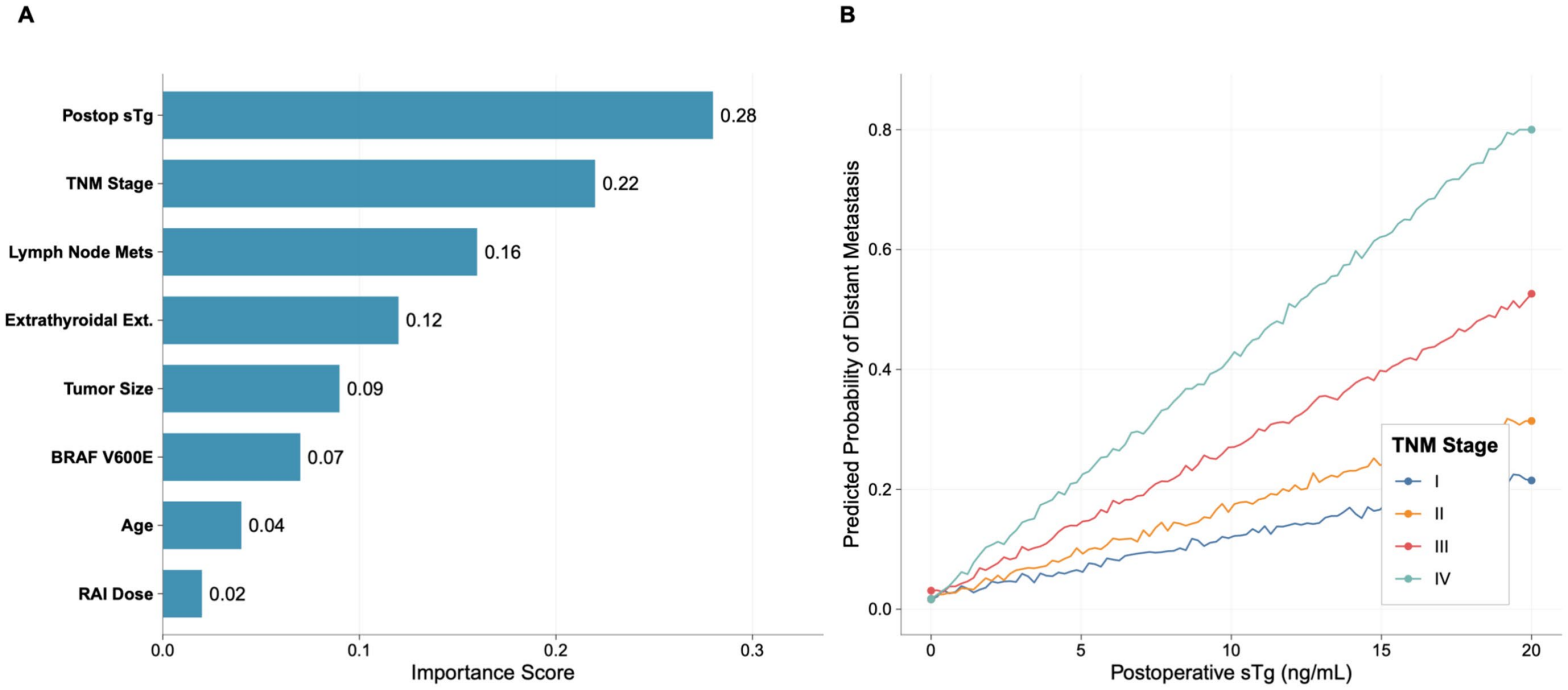
SHAP analysis for model interpretation. **(A)** Feature importance ranking based on SHAP values. **(B)** Interaction effect between postoperative sTg level and TNM stage.

SHAP (SHapley Additive exPlanations) analysis revealed nonlinear relationships between features and predictions (Figure 8). Postoperative sTg showed a nonlinear relationship with the prediction outcome, with a steeper slope at higher sTg values. An interaction effect was observed between postoperative sTg and TNM stage, where the impact of sTg on prediction was more pronounced in patients with advanced TNM stages (III–IV).

### Model stability assessment

**Figure 9:**
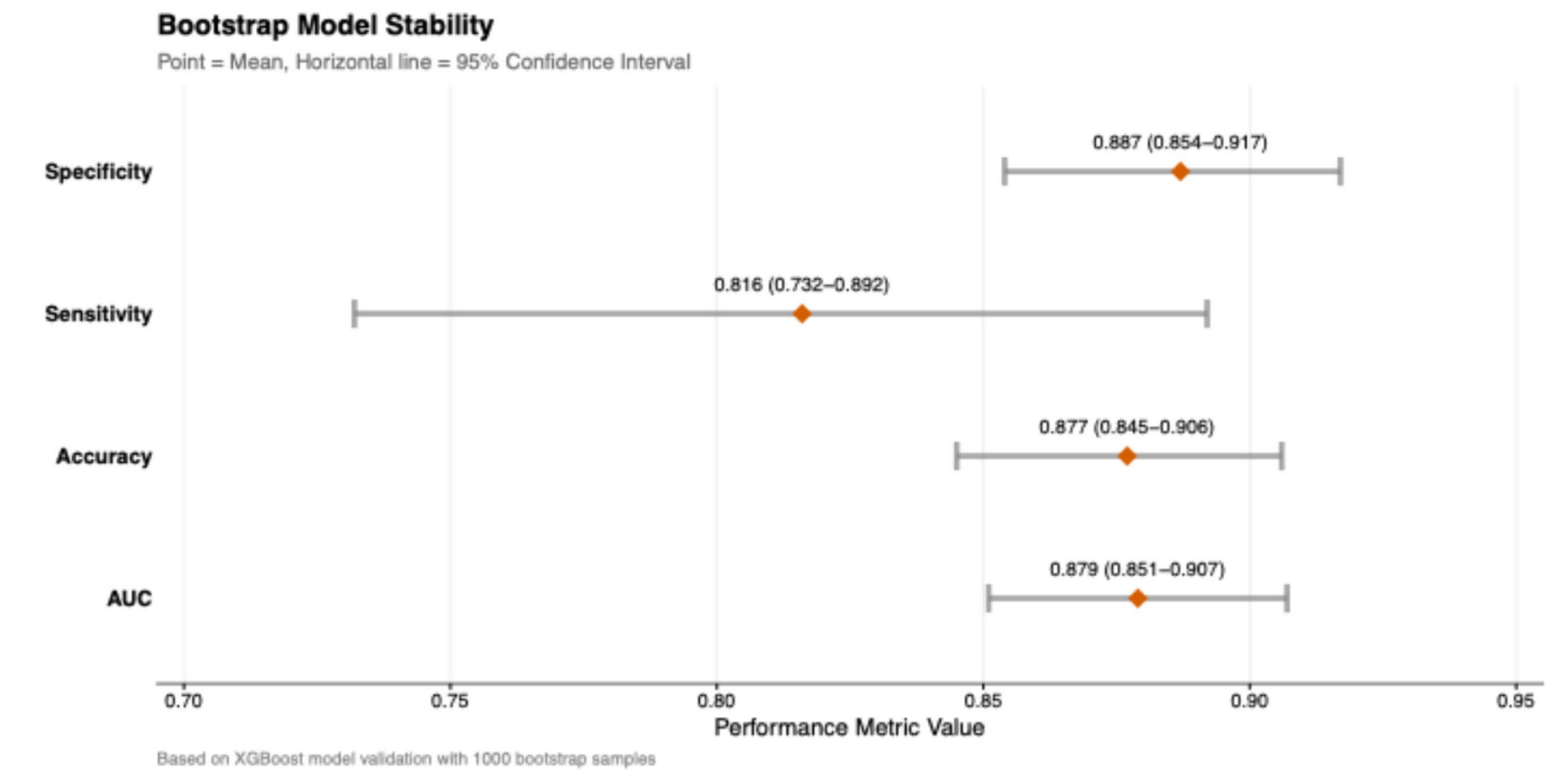
Model stability assessment using Bootstrap validation. Forest plot displaying the mean performance metrics and 95% confidence intervals (CIs) derived from 1,000 bootstrap resamples.

Bootstrap validation with 1,000 resamples was performed to assess the stability of the XGBoost model (Figure 9). The results confirmed the robustness of the model, with mean performance metrics as follows: AUC of 0.879 (95% CI: 0.851–0.907), accuracy of 0.877 (95% CI: 0.845–0.906), sensitivity of 0.816 (95% CI: 0.732–0.892), and specificity of 0.887 (95% CI: 0.854–0.917). The narrow confidence intervals across all metrics indicated that the model maintained high stability and reliability across different subsamples.

## Discussion

In the current study, we developed and validated an interpretable machine learning model based on XGBoost for predicting high-risk distant metastatic recurrence in patients with DTC. This model validated tremendous discriminative capability and effectively graded patients into diverse risk groups with different prognostic outcomes. By integrating clinical, pathological, molecular, and treatment-related variables, the model supplies a comprehensive assessment of metastatic risk that exceeds the capabilities of traditional staging systems.

Consistent with previous reports, postoperative sTg appeared as the most critical predictor in our model (22–24). As a surrogate biomarker for residual thyroid tissue and differentiated tumor cells, sTg levels are directly connected to tumor burden. The crucial advantage of our machine learning method is its ability to capture the nonlinear connection between sTg and metastatic risk. SHAP analysis exposed that the risk gradient steepens markedly at higher sTg levels. Furthermore, this nonlinear relationship suggests that clinical decisions could be more nuanced, moving beyond a single threshold to a risk-adjusted interpretation based on absolute values and their interactions. We found that the prognostic impact of raised sTg was markedly enlarged in patients with advanced TNM stages. The results indicates that an identical sTg level might carry distinct clinical implications depending on the anatomical extent of the disease. TNM stage ranked as the second most vital predictor, confirming its foundational role in DTC prognosis (25, 26). However, the superior performance of our model over TNM staging alone emphasizes the limitations of relying solely on functional factors. While TNM stage delivers critical structural evidence, it fails to account for individual treatment reactions. Our results align with previous investigation implying that traditional staging classifications lack the sensitivity for accurate distant metastasis prediction (27, 28). By integrating lymph node metastasis and extrathyroidal extension—factors exposing aggressive tumor biology (29)—alongside molecular signs, our model offers a more nuanced risk evaluation.

Remarkably, BRAF V600E mutation showed moderate predictive value. While BRAF V600E is commonly associated with aggressive tumor phenotypes and poor consequences in some studies (30, 31), its independent prognostic value is controversial (32). Our model implies that the clinical efficacy of BRAF V600E is amplified when interpreted in conjunction with clinicopathological factors, rather than as a standalone factor. This finding is further corroborated by our sensitivity analysis, which showed that while adding the TERT promoter mutation provided only a marginal performance gain, an integrated framework remains superior to relying on any single molecular marker. This supports the developing agreement that integrative models, which evaluate molecular mutations alongside traditional characters, offer more robust prediction. The clinical advantage of this model is further highlighted by the risk stratification analysis. We recognized three distinct patient cohorts with vastly different trajectories. The high-risk group, characterized by a 64.1% recurrence rate and a 5-year distant metastasis-free survival (DMFS) of only 35.9%, denotes a population that demands aggressive therapeutic strategies. For these patients, the model could guide more aggressive management, such as administering higher cumulative RAI doses (≥200 mCi) or consideration of enrollment in clinical trials for adjuvant targeted therapy. Furthermore, surveillance intensity could be increased to neck ultrasound and serum Tg measurement every 3 months for the first 2 years. Conversely, the low-risk group, with a recurrence rate of only 1.7%, could be managed with de-escalated follow-up protocols, such as annual neck ultrasound and less frequent Tg testing, thereby reducing healthcare costs and patient anxiety. This stratification facilitates the rational allocation of medical resources, reducing unnecessary healthcare costs and alleviating the psychological burden of over-treatment for low-risk patients.

The integration of a comprehensive variable set, including molecular and treatment-related features, ensures a holistic assessment (33, 34). Additionally, the application of XGBoost allowed us to capture complex nonlinear relationships and high-order interactions between variables that are often missed by linear regression models. The identification of specific interaction effects, such as that between sTg and TNM stage, offers clinicians actionable insights into the underlying risk drivers. Despite these strengths, certain limitations must be acknowledged. First, the retrospective, single-center design may introduce selection bias and limit generalizability to broader populations with different demographic characteristics or treatment protocols. Although our propensity score-matched sensitivity analysis confirmed the model’s robustness, external validation in multi-center, prospective cohorts is essential to confirm the model’s transportability. Second, although the current median follow-up has been extended to 72 months, DTC is characterized by indolent behavior. To address the potential for late recurrences, we performed a landmark analysis at the 5-year mark to assess the model’s predictive accuracy for events occurring beyond this period. The model maintained significant discriminative ability (AUC: 0.82) for late recurrence, though extended follow-up remains essential. Third, while the original model was efficient, a sensitivity analysis showed that incorporating TERT promoter mutations offered only a marginal performance gain. This supports our model’s clinical practicality, though future integration of other novel biomarkers like gene expression signatures or circulating tumor DNA could be explored. Finally, as a retrospective study, we cannot exclude potential biases in data collection or missing information. Future research should focus on external validation of this model in diverse ethnic and geographic populations to confirm the model’s universality and transportability. Furthermore, prospective studies should be designed to assess the model’s impact on treatment decision-making and patient outcomes.

In conclusion, we have developed and validated a highly accurate and interpretable machine learning model for predicting high-risk distant metastatic recurrence in DTC. By elucidating complex interactions between key risk factors, this model provides a sophisticated tool that outperforms traditional staging systems. It holds significant promise for guiding personalized treatment intensity and surveillance strategies, ultimately aiming to optimize outcomes for patients with differentiated thyroid cancer. However, widespread clinical application of the model requires further verification of its generalization ability through multi-center prospective studies.

## Data Availability

The original contributions presented in the study are included in the article/Supplementary material, further inquiries can be directed to the corresponding author.
