# Supplementary figures and images for: Development and validation of a machine learning model for predicting high-risk distant metastatic recurrence in differentiated thyroid cancer
Source: Front Med (Lausanne). 2026 Mar 9;13:1790226. doi: 10.3389/fmed.2026.1790226 (PMC13006264; doi:10.3389/fmed.2026.1790226)

# Standard ROC Curves of XGBoost Model

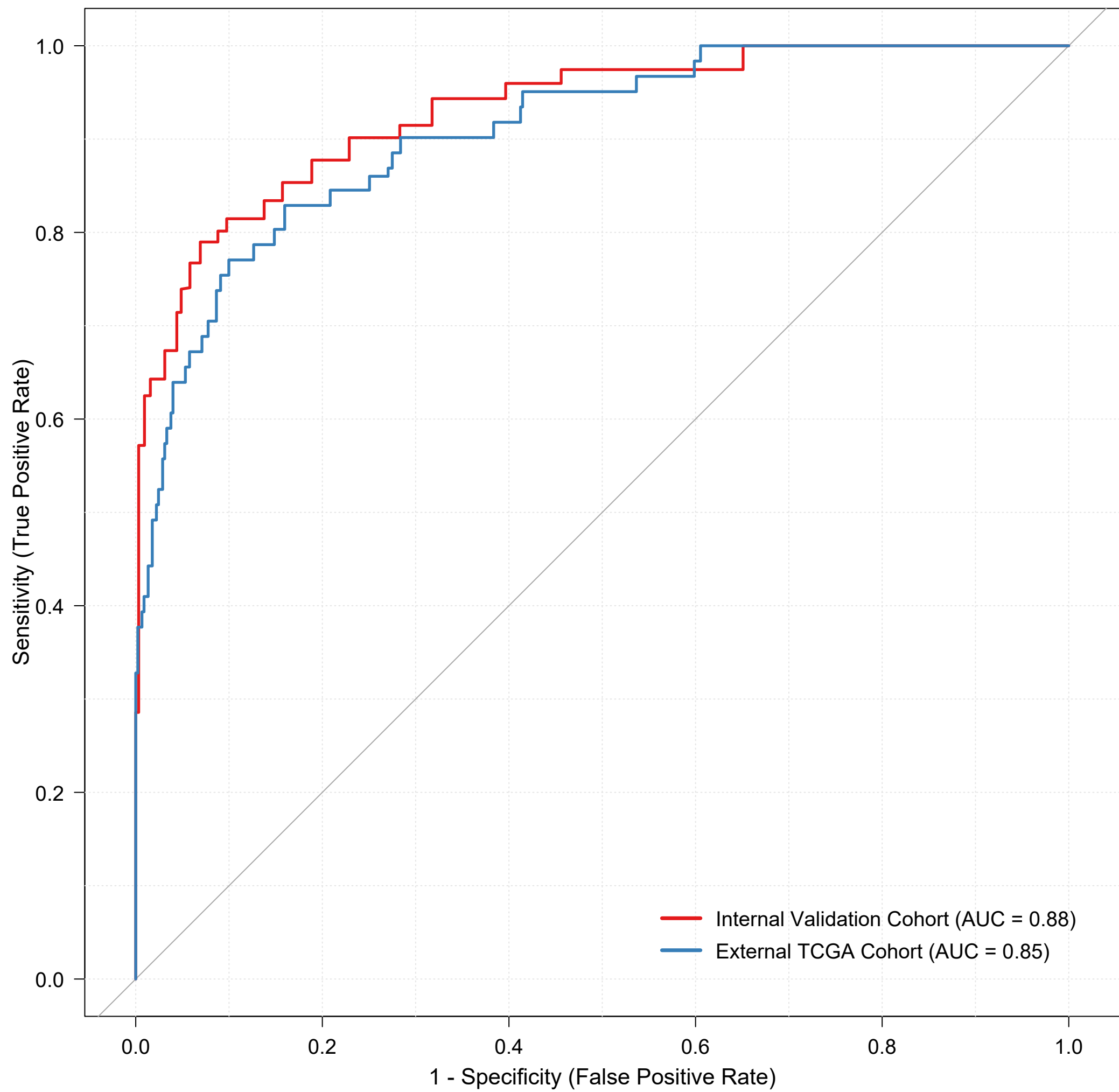

Supplement: Supplementary file 1 [file Image_1.pdf]
